# Homœopathy in Missouri

**Published:** 1895-05

**Authors:** Wm. C. Richardson


### HOMŒOPATHY IN MISSOURI.

Editor of The Homœopathic Physician:—The Missouri Institute of Homœopathy has begun its campaign for recognition by the State government. At a meeting of the Institute in St. Louis in April last, a committee of five was appointed to call upon the State authorities, and urge the granting of such recognition.

The homœopathists will make a strong fight for recognition in the State eleemosynary institutions, more particularly the insane asylums, and for the establishment of a chair of Homœopathy in the State Medical College in the University of Columbia. The committee is composed of Dr. W. C. Richardson and Dr. W. B. Morgan, of St. Louis; Dr. T. H. Hudson and Dr. E. F. Brady, of Kansas City, and Dr. J. H. Ravold, of St. Joseph. They had a long interview with Governor Stone, and have interviewed many members of the Assembly.

The Institute predicates its demands for recognition by the State upon the large number of patrons and practitioners of Homœopathy in Missouri; estimating that the physicians and patrons pay from one-sixth to one-fifth of the entire taxes in Missouri; those in St. Louis paying one-third, and those of Jackson County one-half of the taxes levied in those districts. What the Institute asks, is control of one of the insane asylums of the State, and a chair in the medical department of the State University. The concession with regard to the asylum could be made, without legislation, merely by securing the consent of the governor to appoint a controlling number of homœopaths upon the board of such asylum. As to the desired chair in the university of Columbia, a special order would be asked for directing the curators of the State University to appoint a practitioner of Homœopathy to such chair.

The homœopaths have reached the conclusion that Governor Stone will not wish to assume the responsibility of directing the appointment of a member of their school to a chair in the medical department of the State University, and the special committee of the institute, has prepared a bill for early introduction. Even though the governor might consent to take such action as is desired, the advantage so gained might all be undone by the next State administration.

The matter of securing control of one of the insane asylums of the State, the committee thinks, is entirely dependent upon legislative action, and the committee will proceed with moderation, realizing that it will be necessary for them to conduct a campaign of education, and to guard as thoroughly as possible against attacks by practitioners of the allopathic, or regular school. The body of the homœopathic bill already prepared is as follows:

An act to establish a chair of Homœopathy in the State University.

SECTION 1. The Board of Curators of the State University shall establish and maintain a chair of Homœopathy in the State University of Columbia.

SEC. 2. The instructor, or instructors, in Homœopathy shall be adherents of the homœopathic system of practice, and shall be graduates of medical colleges having a membership in the International Collegiate Committee of the American Institute of Homœopathy.

SEC. 3. The fact that there is no instruction being given in Homœopathy in the State University creates an emergency within the meaning of the Constitution; and, therefore, this act shall take effect and be in force from and after its passage.

Another bill providing for the setting aside of one of the State asylums (insane) for the homœopaths exclusively, is being prepared, and will be introduced soon.

The outcome of this effort to secure a fair distribution of State patronage is anxiously waited for.

Yours very respectfully,

WM. C. RICHARDSON, M. D.

